# Children’s Teeth

**Published:** 1891-11

**Authors:** 


### CHILDREN'S TEETH.

There are very few parents who understand the necessity of keeping a child's first set of teeth in a good, sound condition until the time comes for the appearance of the second teeth. I have seen children with dainty, pretty features, lovely complexions and hair, whose beauty was completely spoiled by a row of black, decayed teeth, sometimes only mere stumps. If a child's teeth be properly cleansed after each meal, or at least twice every day, on rising in the morning and retiring at night, decay may usually be warded off for a long time. The teeth should never be neglected until they are so decayed that extraction is necessary, and the child suffers from that horrible torture, the toothache; besides unnecessary suffering, this neglect often causes serious injury to the permanent teeth. At the first signs of decay or unsoundness of a child's teeth, take the little one to a good dentist and he will probably fill them, making them last much longer, which will be greatly to the advantage of the second set. One cannot have very strong, white teeth unless plenty of bone-making food be taken up by the system. The best food for the teeth consists of all the cereals, pure, rich milk, brown bread, lean meat, vegetables, and fresh fruit. All such things as candy, preserves, cake, pastry, and all other sweets, are absolutely injurious to the teeth, and also harmful to the whole internal organism. Commence cleaning a baby's teeth when they first appear, using borax water and a soft, linen rag. When the child is a year old, a soft little brush may be carefully used, with pure tepid water, and perhaps prepared chalk of the finest and purest kind. Watch carefully the interstices of the teeth, that no food shall find lodgment there.

